# Compassionate Use of Olorofim for Invasive Mold Infections: A Nationwide Observational Study in France

**DOI:** 10.1093/ofid/ofaf750

**Published:** 2025-12-11

**Authors:** V Esnault, C Godet, D Garcia-Hermoso, A Charmillon, P Parize, C Bonnal, A Debourgogne, F Morio, M E Bougnoux, E Dannaoui, A P Bellanger, J P Gangneux, B Sendid, E Cardot, C Melenotte, C Rouzaud, A Lefort, S Colin de Verdiere, O Brugiere, M Tetart, E Eschapasse, A Berceanu, P Tattevin, R Levy, E Faure, F Lanternier

**Affiliations:** Infectious Diseases Department, Henri Mondor University Hospital, Assistance Publique–Hôpitaux de Paris, Créteil, France; Service de Pneumologie et Oncologie Thoracique, Centre Constitutif Maladies Pulmonaires Rares, Sorbonne Université, Assistance Publique–Hôpitaux de Paris, Hôpital Tenon, Paris, France; GRC 40 Sorbonne Study Group for Lung Infectious Diseases(SoLID), Sorbonne Université, Paris, France; Institut Pasteur, Centre National de Référence Mycoses Invasives et Antifongiques, Département de Mycologie, Université Paris Cité, Paris, France; Infectious Diseases Department in Charge of Mobile Infectiology Team, CHRU de Nancy-Brabois,Vandoeuvre-lès-Nancy, France; Necker-Pasteur Center for Infectious Diseases and Tropical Medicine, Necker-Enfants Malades University Hospital, Assistance Publique–Hôpitaux de Paris, Paris, France; Parasitology and Mycology Department, Bichat-Claude Bernard University Hospital, AP-HP, Paris, France; Stress, Immunity, Pathogens Laboratory, Faculty of Medicine, EA7300 Lorraine University, Vandoeuvre-lès-Nancy, France; Parasitology-Mycology Laboratory, CHU Nancy-Brabois, Vandoeuvre-les-Nancy, France; Parasitology and Mycology Department, Nantes University Hospital, UR1155—IICiMed, Nantes Université, Nantes, France; Parasitology and Mycology Laboratory, Necker University Hospital, Paris, France; Biology and Pathogenicity of Fungal Infections Unit, Institut Pasteur, Paris, France; Parasitology and Mycology Laboratory, Necker University Hospital, Paris, France; Faculty of Medicine, Université Paris Cité, Paris, France; Parasitology and Mycology Department, Besançon University Hospital, UMR 6249 CNRS Chrono-Environnement, Univ Franche-Comté, Besançon, France; EHESP, Institut de Recherche en Santé, Environnement et Travail, Inserm UMR-S1085, Univ Rennes, CHU Rennes, Rennes, France; CNR Mycoses Invasives et Antifongiques, Laboratoire Associé AspC, Parasitologie-Mycologie, CHU Rennes, Rennes, France; Laboratory of Mycology and Parasitology, Inserm, U1285-CNRS UMR8576 “Glycobiology in Fungal Pathogenesis & Clinical Applications” Inserm, U1285-CNRS UMR8576, Hospital and University of Lille, Lille, France; Service de Biologie Clinique, Hôpital Foch, Suresnes, France; Necker-Pasteur Center for Infectious Diseases and Tropical Medicine, Necker-Enfants Malades University Hospital, Assistance Publique–Hôpitaux de Paris, Paris, France; Necker-Pasteur Center for Infectious Diseases and Tropical Medicine, Necker-Enfants Malades University Hospital, Assistance Publique–Hôpitaux de Paris, Paris, France; Department of Internal Medicine, Beaujon Hospital, AP-HP, Clichy, France; Centre de Transplantation Pulmonaire et CRCM, Hôpital Foch, Suresnes, France; Centre de Transplantation Pulmonaire et CRCM, Hôpital Foch, Suresnes, France; Department of Infectious Diseases, Hospital of Tourcoing, Tourcoing, France; Pneumology Department, CHU Lille, Lille, France; Service de Pneumologie, L’Institut du Thorax, Hôpital Guillaume et René Laënnec, Centre Hospitalier Universitaire de Nantes, Nantes, France; Hematology Department, Besançon Hospital, Besançon, France; Infectious Diseases and Intensive Care Unit, Pontchaillou University Hospital, Rennes, France; Pediatric Immunology-Hematology and Rheumatology Unit, Necker Hospital for Sick Children, AP-HP, Paris Cité University, Imagine Institute, Paris, France; Infectious Diseases Department, CNRS, Inserm, Institut Pasteur Lille, U1019-UMR 9017-CIIL (Center for Infection and Immunity of Lille), CHU Lille, Université de Lille, Lille, France; Institut Pasteur, Centre National de Référence Mycoses Invasives et Antifongiques, Département de Mycologie, Université Paris Cité, Paris, France; Necker-Pasteur Center for Infectious Diseases and Tropical Medicine, Necker-Enfants Malades University Hospital, Assistance Publique–Hôpitaux de Paris, Paris, France

**Keywords:** antifungal agents, fungal diseases, fungal infections, invasive pulmonary aspergillosis, olorofim

## Abstract

**Background:**

The increasing incidence of resistant invasive mold infections (IMIs) has highlighted the need for novel antifungal agents. Olorofim, a first-in-class orotomide, has shown promising efficacy in a recent phase II study, but clinical data remain limited.

**Methods:**

We conducted a retrospective multicenter cohort study in France, including all patients who received olorofim under compassionate use for proven or probable non-Mucorales IMIs. Eligible patients had IMIs refractory to or intolerance to standard antifungals or no effective treatment options. Efficacy was defined as mycological and clinical control of infection; safety was also assessed.

**Results:**

Between January 2020 and December 2023, 17 patients (median age, 39 years) received olorofim. Underlying conditions included primary immunodeficiency (n = 4) and lung transplantation (n = 5). Sites of infection included the lung (88.2%) and the central nervous system (23.5%), with 5 cases of disseminated disease. In total, 23 strains were identified, mostly *Aspergillus fumigatus* (34.8%) and *Microascus* spp (13%). The median duration of prior antifungal therapy was 9.1 months. Olorofim was used primarily for refractory IMIs (70.6%) and in combination with other antifungals in 82.4% of cases. Olorofim minimum inhibitory concentrations were ≤0.5 mg/L in the 8 available isolates. Among 15 evaluable patients, 5 (33.3%) achieved clinical and mycological success, 7 (46.7%) had partial responses, and 3 (20%) experienced treatment failure. The 3-month mortality rate was 29.4% (5 of 17). No severe adverse events were reported.

**Conclusions:**

Olorofim exhibited potential efficacy and good tolerability in patients with refractory IMIs. Further data from ongoing clinical trials are needed to confirm these findings.

## Teaser

This first report of olorofim use in real-world clinical practice highlights the promising efficacy and good tolerability of olorofim in patients with invasive mold infections unresponsive to standard therapies, supporting its potential role in difficult-to-treat cases.

The incidence of invasive mold infections (IMIs) has increased over the past 2 decades, along with the emergence of new populations at risk. The use of broad-spectrum antifungal prophylaxis in these individuals has led to the emergence of previously uncommon pathogens, such as *Scedosporium* spp and *Lomentospora prolificans*, as well as cryptic species of *Aspergillus* spp, all characterized by reduced susceptibility to existing antifungal agents and associated with poor clinical outcomes [1, 2]. Similarly, the widespread use of azole fungicides in agriculture has driven the emergence of multiresistant *Aspergillus fumigatus* strains, leading to therapeutic challenges and raising serious public health concerns across several European countries [3].

To address these challenges, the development and evaluation of new antifungal therapies has become imperative. Among novel agents, olorofim stands out as the only representative of the orotomide class. Olorofim acts as a reversible and selective inhibitor of fungal dihydroorotate dehydrogenase, an enzyme involved in *de novo* pyrimidine biosynthesis, thus preventing the growth of multiple ascomycetous molds [4]. This unique mechanism is believed to account for the sustained efficacy of olorofim against numerous molds, including strains with limited susceptibility, except for Mucorales (no activity) and some *Fusarium* species (reduced activity) [5]. However, most data come from *in vivo* models of invasive mold diseases [6, 7]. Recently, a single-arm, open-label, phase II clinical study of olorofim for the treatment of IMIs in patients with limited treatment options has reported efficacy against highly resistant organisms and good tolerability in prolonged courses [8]. Additional clinical data come from case reports showing promising results [9–11]. A phase III randomized clinical is ongoing to compare olorofim with liposomal amphotericin B in patients with proven or probable invasive aspergillosis either refractory to or unsuitable for azole therapy (NCT05101187).

From 2020 to 2023, olorofim was accessible in France for compassionate use following the same inclusion criteria as in the phase II trial (ie, proven or probable invasive aspergillosis without therapeutic options or proven infection due to an olorofim-susceptible mold without alternative therapeutic option). The objective of the present study was to describe the characteristics and outcomes of the first patients treated with olorofim in real-life clinical setting in France.

## METHODS

We performed a retrospective multicenter national cohort study including every patient prescribed olorofim as a compassionate treatment for proven or probable IMIs between 1 January 2020 and 31 December 2023. Four cases have already been described elsewhere but are again reported here to provide a comprehensive view [9, 10]. Olorofim introduction criteria were proven non-Mucorales mold infection refractory to standard antifungal agents, intolerance to such agents, or IMIs with no effective antifungal therapy available. IMIs were required to meet criteria for proven (any fungal pathogen) or probable (*Aspergillus* only) fungal diseases as defined by the European Organization for Research and Treatment of Cancer and the National Institute of Allergy and Infectious Diseases Mycoses Study Group (EORTC/MSG) [12]. Patients enrolled in blinded trials and those who did not ultimately receive olorofim despite prescription were excluded from the analysis.

Our primary outcome was efficacy as defined by mycological and clinical control of IMIs using the EORTC/MSG criteria [13], which, though developed for clinical trial settings, provide a standardized approach for evaluating responses to antifungal therapy. A minimum duration of 15 days of olorofim therapy was required to assess its efficacy. Secondary outcomes included tolerance and safety of olorofim, evaluated by collecting drug-related adverse events and premature treatment discontinuations. Patients were followed up until death or the end of the study period on 31 December 2024.

Olorofim was provided by F2G as 30-mg tablets for oral administration. The recommended dosing regimen was a loading dose of 150 mg every 12 hours on day 1, followed by 90 mg every 12 hours, as suggested by F2G. Dosing adjustments were permitted based on drug interactions.

Clinical and laboratory data were retrospectively collected from patients' electronic health records. The minimum inhibitory concentrations (MICs) of olorofim and standard antifungals were determined by the National Reference Center for Invasive Mycoses and Antifungals (CNRMA), Institut Pasteur, following the European Committee on Antimicrobial Susceptibility Testing (EUCAST) microdilution method [13]. For *A fumigatus* strains not submitted to the CNRMA, the MIC of voriconazole was collected, using the Etest gradient strip method performed by the local laboratory.

Informed consent was obtained from the patient or, if the patient was deceased at the time of data collection, from the legally authorized representative or next of kin, in accordance with applicable ethical guidelines. The study design was approved by the Assistance Publique - Hôpitaux de Paris - Centre ethical committee (institutional review board no. 00011928).

## RESULTS

### Patient Characteristics

Between 1 January 2020 and 31 December 2023, 19 patients were prescribed olorofim in France for compassionate treatment of IMIs with limited or no therapeutic option. Two individuals were excluded from this analysis as they did not receive olorofim despite prescription: 1 patient improved and 1 died before delivery. The remaining 17 patients were included in the study. Their underlying diseases are shown in Table 1: 4 (23.5%) had a primary immunodeficiency, including 3 with chronic granulomatous disease and 1 with *caspase recruitment domain family member 9* (CARD9) deficiency, and 7 (41.2%) were solid organ transplant recipients, mostly lung transplant recipients (5 of 17 [29.4%]); among those, 3 had a history of chronic lung allograft dysfunction prior to IMIs.

**Table 1.** Clinical Characteristics of Patients and Evolution Under Olorofim

Patient	Age, y; Sex	Main Underlying Diseases	Pathogen	Site of Infection	Standard EUCAST AF MIC, mg/L	Time on Prior AF, mo	Prior Surgery	Indication for Olorofim	Olorofim MIC, mg/L	Concomitant AF Therapy	Outcome at Analysis
1	66; M	Lung adenocarcinoma	*Aspergillus fumigatus*	Lung	NP; Etest for VCZ, 4	11.2	Pleurostomy	Refractory infection	…	CAS + inhaled AMBL	Death in <15 d
2	23; M	HSCT for ALL, complete remission; chronic digestive GVHD	*Aspergillus nidulans,* *A fumigatus*	Lung	NP; *A fumigatus*: Etest for VCZ, 0.25	5.1	…	Refractory infection	…	…	Death in <15 d
3^a^	17; M	None; trauma	*Microascus melanosporus*	Lung	AMB, CAS, and MFG,>4; ITZ and PCZ, >8; VCZ, 8; ISZ, 4; TBF, 0.25	0.8	…	Refractory infection	0.25	TBF	Success (6-wk therapy)
4	17; M	None; drowning	*Scedosporium apiospermum*	Disseminated (lung and CNS)	…	7.1	Brain abscess drainage	Refractory infection	…	…	Success (9-mo therapy)
5^a^	60; M	Lung transplantation, CLAD	*Microascus cirrosus, Aspergillus flavus*	Lung	*M. cirrosus*: AMB, ISZ, and CAS, >4; ITZ and PCZ, >8; VCZ, 4 *A flavus*: AMB, >4; ITZ and PCZ and CAS, 0.5; VCZ, 1; ISZ, 4	26.4	…	Refractory infection	*M cirrosus*: 0.5; *A flavus*: 0.016	…	Success (12-mo therapy)
6	57; M	Combined liver and lung transplantation	*S apiospermum,* *Aspergillus calidoustus,* *Aspergillus tubingensis*	Lung	*S apiospermum*: AMB, >4; ITZ and PCZ, 1; VCZ, 0.5; ISZ, 0.25; CAS, 2	26.3	…	Refractory infection	*S apiospermum*: 0.06	TBF + VCZ + inhaled CAS > PCZ + inhaled CAS	Success (21-mo therapy)
7^a^	64; M	Lung transplantation, CLAD	*M cirrosus*	Lung	AMB, ITZ, VCZ, and CAS, >32; ISZ, 1.5	4.1	…	Refractory infection	0.06	TBF + ISZ	Success (3.5-mo therapy); death^d^
8	22; M	Craniopharyngioma resection	*A fumigatus*	CNS	…	9.1	…	Stable infection with intolerance to AF	…	ISZ + intrathecal AMBL > PCZ	PR, ongoing with PCZ
9	73; F	Bronchiectasis (α-1 antitrypsin deficiency)	*A fumigatus,* *Scedosporium aurantiacum*	Lung	NP; *A fumigatus*: Etest for VCZ, 4	4	…	Refractory infection	…	CAS	PR, ongoing with CAS
10	37; M	Sharp syndrome, interstitial lung disease	*A fumigatus*	Lung	NP; Etest for VCZ, 0.19	29.5	…	Refractory infection	…	PCZ	PR, ongoing
11	39; M	CARD9 deficiency	*A fumigatus*	Disseminated (lung and CNS)	AMB, 1; ITZ, VCZ, ISZ, and CAS, 0.5; PCZ, 0.125	17.2	…	Refractory infection	…	ISZ > ISZ + CAS > CAS	PR, ongoing with CAS
12^b^	14; M	CGD	*A fumigatus*	Lung	AMB, 0.25; ITZ, >8; VCZ, 4; PCZ and CAS, 0.5; ISZ, >4 (TR34/L98H^+^)	15.2	Lobectomy	Stable infection with intolerance to AF	0.03	CAS	PR, ongoing
13	15; M	CGD	*Rasamsonia aegroticola*	Lung	AMB and ISZ, >4; ITZ and VCZ, >8; PCZ, 1; CAS, 0.5; MFG, 0.007; 5FC, 0.023^c^	11.1	Parietal abscess drainage	Refractory infection	…	MFG + 5FC	PR, ongoing with MFG + 5FC
14	57; F	Liver transplantation	*A fumigatus*	Disseminated (kidney, pericardium, and lung)	AMB, 0.5; ITZ and VCZ, >8; PCZ, 1; ISZ >4; CAS, 0.5; MFG, 0.007	11.2	Pericardial abscess drainage	Stable infection with intolerance to AF	…	CAS	Relapse, ongoing
15	36; M	CGD, liver transplantation	*Aspergillus udagawae,* *Aspergillus latus*	Disseminated (lung and CNS)	*A latus*: AMB, and ITZ, 1; VCZ, PCZ, and ISZ, 0.5; CAS, 0.25; MFG, <0.008 *A udagawae*: AMB, >4; ITZ, 0.5; VCZ, 2; PCZ, 0.25; ISZ, 2; CAS, 0.25; MFG, 0.007	6.0	…	Refractory infection	*A udagawae*: 0.007	VCZ > CAS	Mycological failure, death
16	60; M	Cystic fibrosis, lung transplantation, CLAD	*Lomentospora prolificans*	Disseminated (knee, skin, sinus, and fungemia)	AMB, ISZ, CAS, and MFG, >4; ITZ, 16; VCZ and PCZ, >8	0.2	…	No effective AF	0.06	TBF + VCZ	Mycological failure, death
17	50; F	Lung transplantation	*Scopulariopsis* spp	Lung	AMB, VCZ, PCZ, and ISZ, >32; MFG, 0.032	0.5	…	No effective AF	…	MFG + VCZ	Mycological failure, death

Abbreviations: 5FC, 5-fluorocytosine; AF, antifungal; ALL, acute lymphoblastic leukemia; AMB, amphotericin B; CARD9, *caspase recruitment domain family member 9* ; CAS, caspofungin; CGD, chronic granulomatous disease; CLAD, chronic lung allograft dysfunction; CNS, central nervous system; EUCAST, European Committee on Antimicrobial Susceptibility Testing; F, female; GVHD, graft-vs-host disease; HSCT, hematopoietic stem cell transplantation; ISZ, isavuconazole; ITZ, itraconazole; M, male; MFG, micafungin; MIC, minimum inhibitory concentration; NP, not performed; PCZ, posaconazole; PR, partial response; TBF, terbinafin; VCZ, voriconazole.

^a^Cases published in [9].

^b^Case published in [10].

^c^The Etest method was used for the 5FC MIC.

^d^Death of cardiovascular origin not related to IMI.

### Fungal Infection Characteristics

The site of infection was predominantly the lung (15 of 17 [88.2%]) or the central nervous system (CNS) (4 of 17 [23.5%]). Disseminated infection was reported in 5 patients (29.4%) (Table 1). A total of 23 fungal strains were identified, mostly *Aspergillus,* including *A fumigatus* (n = 8 [34.8%]) and 6 other species (*Aspergillus flavus*, *Aspergillus calidoustus*, *Aspergillus latus*, *Aspergillus tubingensis*, *Aspergillus nidulans,* and *Aspergillus udagawae*). Other fungi included *Microascus* spp (n = 3 [13%]), *Scedosporium* spp (n = 3), *L prolificans*, *Rasamsonia aegroticola,* and *Scopulariopsis alboflavescens.* Mixed IMIs caused by ≥2 fungi were reported in 5 of 17 patients (29.4%) (Table 1).

The MICs of standard antifungal agents against those pathogens are shown in Table 1. Four *A fumigatus* strains were azole resistant (4 of 7 [57%]; data missing in 1). Rare molds, such as *Microascus* spp, *Scopulariopsis* spp, *L prolificans,* and *Rasamsonia* spp, displayed high MICs for azoles and polyenes, with various susceptibility to echinocandins.

All patients received prior antifungal therapy for the IMI of interest, with a median of 2 therapeutic regimens (range, 1–6) before olorofim initiation. Azoles were prescribed in 16 of 17 individuals (94.1%), mostly voriconazole (n = 12), followed by isavuconazole (n = 3) and itraconazole (n = 1). First-line azole therapy in combination with another antifungal was reported in 3 cases: (1) voriconazole and caspofungin for disseminated lung and CNS infection due to *A latus* and *A udagawae*, (2) voriconazole and terbinafine for disseminated *L prolificans* infection, and (3) isavuconazole and inhaled liposomal amphotericin for invasive lung infection with 2 cryptic *Aspergillus* species and *Scedosporium apiospermum*. Overall, patients had been on prior antifungal therapy for a median duration (interquartile range [IQR]) of 9.1 (4.1–15.2) months before olorofim therapy. Five individuals underwent surgery before olorofim therapy (Table 1).

### Olorofim Therapy

Olorofim was started within 15 days of IMI diagnosis in 2 patients for whom no effective antifungal therapy was available: 1 with disseminated lomentosporiosis and 1 with *Scopulariopsis* lung infection. Other reasons for olorofim introduction included refractory IMI in 12 patients (70.6%) and intolerance to antifungal treatment despite stable infection in 3 (17.6%) (Table 1).

When available (n = 8), olorofim MICs were ≤0.5 mg/L. Notably, olorofim MICs against *L prolificans* and azole-resistant *A fumigatus* harboring the TR34/L98H mutation were 0.06 and 0.03 mg/L, respectively. Olorofim therapy was mostly administered as a combination therapy (n = 14 [82.4%]) (Table 1). Eight individuals (47.1%) received ≥2 other antifungal agents. No adjuvant surgery was performed after olorofim initiation.

Two patients died within 15 days after olorofim introduction and were excluded from the efficacy analysis. Mycological failure was reported in 3 of 15 patients (20%) who died with refractory IMIs, including (1) disseminated lomentosporiosis with fungemia, fungal arthritis, cutaneous lesions, and sinusitis in a lung transplant recipient; (2) *Scopulariopsis* lung infection in a lung transplant recipient; and (3) lung and CNS aspergillosis caused by *A udagawae* and *A latus* in a liver transplant recipient with chronic granulomatous disease. In these 3 patients, the median time between diagnosis and olorofim introduction was 15 days (range, 7–181 days). All 3 received concomitant antifungal therapy with voriconazole.

A successful outcome leading to olorofim discontinuation was reported in 5 patients (Table 1). Among these, 2 patients were immunocompetent, 1 with posttraumatic *Microascus melanosporus* lung infection and 1 with postdrowning *S apiospermum* lung and cerebral infection who underwent neurosurgery before olorofim therapy. The other 3 patients were lung transplant recipients with lung IMIs. Among the 5 patients, the median duration of olorofim therapy (IQR) was 9 (3.5–12) months. Six patients (40%) achieved partial clinical and mycological response: 4 with aspergillosis, including lung, CNS, and disseminated infections; 1 with *Scedosporium aurantiacum* and *A fumigatus* lung infection; and 1 with *Rasamsonia* lung infection. Olorofim therapy was ongoing at the time of analysis in all 6 individuals. Combination antifungal therapy was reported in 4 of 6 (Table 1). Relapse of invasive pulmonary aspergillosis was reported in 1 patient 5 months after olorofim discontinuation, confirmed by 2 positive *A fumigatus* blood polymerase chain reaction results; a partial response was achieved after resumption of olorofim. The median duration of olorofim (IQR) in patients pursuing therapy was 26.3 (19.8–30.4) months. Overall, the 3-month all-cause mortality rate was 29.4% (5 of 17).

Finally, no severe adverse event related to orolofim was reported. One patient experienced persistent nausea during olorofim and voriconazole therapy without significant weight loss, leading to poor adherence and olorofim discontinuation. Reintroduction of olorofim in combination with caspofungin instead of voriconazole resulted in improved tolerance. Mild elevation of alanine aminotransferase levels was reported in 1 individual (3 times the upper limit of normal) and resolved with olorofim dosage adjustment. Asymptomatic lability of the international normalized ratio during antithrombotic therapy was noted in 1 patient and then stabilized without dose modification. Among solid organ transplant recipients, modification of immunosuppressive therapy was reported in 4 of 5 (with missing data in 2 patients) and consisted of decreasing the dosage or discontinuing immunosuppressive drugs in 3 individuals with uncontrolled IMIs and increasing the tacrolimus dosage due to low plasmatic troughs following olorofim introduction in another.

## DISCUSSION

As a novel antifungal agent, olorofim has emerged as a promising option against the many challenges of escalating resistance and the rising incidence of rare molds. Thus far, clinical data on olorofim use to treat IMIs remained scarce. The recent 200-patient phase II trial reported efficacy and good tolerability of olorofim in patients with invasive fungal infections with few or no treatment options [8]. Here, we describe the characteristics and outcome in 17 patients who received olorofim as compassionate therapy for IMIs in France from 2020 to 2023.

Our cohort reflects the heterogeneity and complexity of IMI management. Most patients were immunocompromised, with a history of lung transplantation in 29.4% and primary immune deficiency in 23.5%. Chronic allograft dysfunction was frequently reported among lung transplant recipients, suggesting increased immunosuppression related to rejection treatment.

*A fumigatus* was the most frequently isolated pathogen, accounting for a third of cases, often in the context of coinfection. Other non-*fumigatus Aspergillus* species with elevated MICs for standard antifungal agents were frequent. We also reported rare molds like *L prolificans*, *R aegroticola*, *Microascus* spp, *Scopulariopsis* spp, and *Scedosporium* spp, for which treatment options are limited and based on expert recommendations. All patients in our cohort had received prior antifungal therapy, after extensively longer courses than reported in the phase II trial (median, 9.1 vs 2.5 months, respectively [8]), usually involving a combination of drugs, including intravenous formulations. Extensive prior exposure raises concerns regarding the need for therapeutic drug monitoring, potential drug interactions, tolerance, cost, and emergence of breakthrough fungal infection. Conversely, olorofim, with its oral formulation and notably low MIC, represents a promising alternative. Consistent with the literature, olorofim demonstrated MICs <0.1 mg/L against several *Aspergillus* species and rare molds, even among patients exposed to many antifungals, such as those with chronic granulomatous disease [4, 6].

The 3-month all-cause mortality rate in our cohort was 29.4%, compared with 16.3% in the phase II trial [8]. This difference is likely related to the high comorbidity and immunosuppressive burden of our population, in whom olorofim was mainly prescribed as compassionate therapy for refractory IMIs. Notably, efficient control of the infection was achieved in almost 80% of individuals. Success was reported in approximately a third of cases, including 3 cases of *Microascus* bronchopulmonary infection, which have been more extensively described elsewhere [9]. While 2 patients were immunocompetent, the others were lung transplant recipients, suggesting that a successful outcome might be achieved despite ongoing immunosuppression. Importantly, the median duration of olorofim therapy among responders was 9 months, and 3 patients received concomitant antifungal therapy, thus limiting the interpretation of olorofim specific efficacy.

Partial response with ongoing olorofim therapy at the time of analysis was also frequently reported. Similar results were observed in the phase II trial, with striking differences in favorable global response at day 42 whether or not stable disease was considered as success or not: 75.2% (95% confidence interval, 68.7%–81.0%) versus 28.7% (22.6%–35.5%), respectively [8]. These findings raise important considerations: while these cases would be regarded as therapeutic failures in clinical trials with short follow-up periods, stabilization of the infection may represent a significant achievement when managing complex IMIs in highly immunocompromised patients. Whether partial response is related to host factors, fungal characteristics, or the necessity for antifungal combinations remains to be established. Notably, 1 patient experienced relapse after discontinuing olorofim and achieved partial response after its reintroduction, suggesting that olorofim may act as a suppressive therapy in some cases.

Importantly, despite the extended duration of olorofim treatment in our cohort, it was well tolerated, with no severe adverse events reported and only a single case of mild aminotransferase elevation. As olorofim is metabolized by several cytochrome P450 isoenzymes and acts as a weak inhibitor of CYP3A4 [14], potential drug interactions should be anticipated and carefully managed, though available data suggest a predictable and limited impact on other drugs, such as anticalcineurin inhibitors [8]. To date, therapeutic drug monitoring of olorofim is not yet routinely available but may be performed by the manufacturer as needed.

Treatment failure despite olorofim was reported in 3 patients with refractory IMIs. Several factors may have contributed to this outcome. The patient with invasive lomentosporosis had fungal knee arthritis, representing a significant fungal burden that was not surgically addressed due to the patient’s overall condition, potentially limiting antifungal efficacy. In the *Scopulariopsis* case, discontinuing immunosuppressive drugs resulted in acute graft rejection, requiring high-dose corticosteroids alongside antifungal treatment. All 3 patients with treatment failure received concomitant voriconazole therapy. It has been suggested that azoles may antagonize olorofim activity against *A* fumigatus *in vitro* by up-regulating the pyrimidine biosynthesis pathway [15]. However, we reported complete or partial mycological responses among 5 other patients with concomitant azole therapy. Further studies are needed to elucidate the interaction between olorofim and azoles. Other antifungal combinations involving olorofim also remain to be evaluated in clinical trials.

Our study is limited by its retrospective design and our small, heterogeneous population, which prevented us from identifying factors associated with the success or failure of olorofim. In addition, antifungal susceptibility testing was not available for all isolates and may have influenced treatment modifications, though the clinic relevance of olorofim MICs remains to be fully established. Routine therapeutic drug monitoring of olorofim was not available either. However, along with the recent phase II trial, our study provides a description of olorofim therapy, and the reported experience provides additional guidance that highlights the many challenges of managing IMIs and evaluating new antifungals.

In conclusion, in this retrospective multicentric study, we present the first real-life data of olorofim use against IMIs. Despite a limited sample, our findings suggest that olorofim exhibits low MICs against molds resistant to standard antifungal agents and that prolonged olorofim therapy is an effective and well-tolerated option for refractory IMIs, including *Microascus* spp.
